# PPIs therapy has a negative impact on the clinical outcomes of advanced SCLC patients treated with PD-L1 inhibitors

**DOI:** 10.1186/s12890-023-02754-4

**Published:** 2023-11-11

**Authors:** Sisi Zhang, Jing Tian, Xinwei Wang, Chengxin Liu

**Affiliations:** 1 Shandong First Medical University, Shandong Academy of Medical Sciences, Jinan, Shandong 250062 China; 2 Department of Radiation Oncology, Shandong Cancer Hospital and Institute, Shandong First Medical University, Academy of Medical Sciences, Jinan, Shandong 250117 China; 3 Department of Radiation Oncology, Jinan Zhangqiu District People’s Hospital, Jinan, Shandong 250200 China; 4 Department of Intensive Care Medical Center, Shandong Public Health Clinical Center, Shandong University, Jinan, Shandong 250013 China

**Keywords:** Immune checkpoint inhibitors, PD-L1 inhibitors, Immunotherapy, Proton pump inhibitors, Progression free survival, Overall survival

## Abstract

**Background:**

Programmed death-ligand 1 (PD-L1) inhibitors has emerged as a first-line therapeutic strategy for advanced small cell lung cancer (SCLC), which can stimulate T-cell activation, thereby preventing tumor avoidance of immunologic surveillance, whereas, proton pump inhibitors (PPIs) can play an important role in regulating immune function. This study assessed whether the concomitantly use of PPIs affected outcomes of immunotherapy in advanced SCLC.

**Methods:**

Data from advanced SCLC patients who firstly treated with PD-L1 inhibitors between July 2018 and February 2021 was retrospectively analyzed. The impact of concomitant medications (especially PPIs) on objective response rate, progression-free survival (PFS) and overall survival (OS) were evaluated.

**Results:**

Of 208 patients, 101 received immunotherapy concomitant PPIs. The median PFS of patients receiving PPIs (6.6 months) were significantly shorter than those without PPIs (10.6 months), and so was OS. There was associated with a 74.9% increased risk of progression and 58.3% increased risk of death. Both first-line and post-first-line immunotherapy, patients treated PPIs had poorer PFS.

**Conclusion:**

PPIs therapy has a negative impact on the clinical outcomes of advanced SCLC patients treated with PD-L1 inhibitors.

**Supplementary Information:**

The online version contains supplementary material available at 10.1186/s12890-023-02754-4.

## Background

Small cell lung cancer (SCLC) is an aggressive and relatively rare subtype of lung cancer, accounts for 13-15% of all lung cancer patients [1, 2]. However, due to its hidden onset and insufficient early screening, about 70% of SCLC patients were in the extensive period when they were first diagnosed [3, 4]. In recent years, the emergence of immune checkpoint inhibitors (ICIs), including programmed death ligand 1 (PD-L1) inhibitors and programmed death receptor 1 (PD-1) inhibitors, has changed the survival outcome of patients with aggressive extensive SCLC [5]. Many clinical trials of ICIs were conducted in patients with advanced SCLC. Based on the phase 3 IMpower133 [6, 7] and CASPIAN study [8, 9], the combination of platinum-etoposide chemotherapy with atenlibizumab or duvarizumab (chemoimmunotherapy) has become the new standard for first-line treatment of patients with extensive stage SCLC [5, 9, 10].

At present, many clinical trials have pointed out that the “abuse” of PPI affected the efficacy of anti-tumor treatment, both in chemotherapy, targeted therapy and immunotherapy [11–13]. The proton pump inhibitors (PPIs) were used in 20–33% of the tumor patients to relieve the upper gastrointestinal symptoms, including acid reflux, vomiting, and upper abdominal discomfort during the treatment [14, 15]. Data pooled from phase II POPLAR (NCT 01903993) and phase III OAK (NCT 02008227) trials showed that OS and PFS were significantly shorter in NSCLC patients who received PPIs, within the atezolizumab population (9.6 versus 14.5 months, 1.9 versus 2.8 months, *P* = 0.001) [16–19].

Similarly, a meta-analysis suggested that concomitant PPIs use is significantly associated with low clinical benefit in ICIs treatment, revealing a significantly reduced PFS and OS in advanced cancer patients receiving ICIs who are also exposed to PPIs [20]. Concomitant antacid use could modify the activity of ICIs in NSCLC patients [21]. Furthermore, S. Buti et al. [22, 23] identified the cumulative poor prognostic role of concomitant medications, namely, corticosteroids, antibiotics and PPIs, on the clinical outcome of patients with advanced cancer treated with ICIs. After the case-control random matching study of patients with NSCLC receiving first-line pembrolizumab or chemotherapy found that baseline medications are more likely to affect ICI’s efficacy rather than the cytotoxic mechanism of action of chemotherapy. The prognostic stratification in terms of PFS and OS was significantly more pronounced among the pembrolizumab-treated patients. These prior evidences support the hypotheses that PPIs may be strongly associated with altered ICIs efficacy.

Corticosteroids are broadly used as premedication for chemotherapy regimens and are frequently used to alleviate pain or dyspnea, to stimulate appetite [24]. However, corticosteroids have anti-inflammatory and immunosuppressive effects which was significantly related to worse clinical outcomes with cancer immunotherapy [25].Indeed, ORR, PFS and OS were significantly worse in NSCLC patients treated with baseline steroid treatment in symptom relief as found in the report by Ricciuti et al. [26].

As patients age, the incidence of concomitant diseases such as hypertension and cardiovascular disease, as well as related multi drug treatment, increases. It is known that there is a certain relationship between aspirin and cancer prevention/progression, but there is little research in the context of cancer immunotherapy [27, 28]. Concomitant medications including PPIs, corticosteroids, and other drugs have been postulated to exert immune-modulatory effects within the tumor microenviroment, thus affecting clinical outcomes from ICI therapy. However, less information is available on the effect of concomitant medications on ICIs efficacy with SCLC. This study aimed to evaluate whether the long-term use of concomitant medications had a negative impact on the clinical outcomes of PD-L1 inhibitors in patients with advanced SCLC.

## Methods

### Study design

We retrospectively identified and included 208 patients with advanced or relapsed SCLC who started anti-PD-L1 based therapies between July 2018 and February 2021 at the Affiliated Cancer Hospital of Shandong First Medical University, China. Patients received anti-PD-L1 inhibitors such as 1500 mg durvalumab or 1200 mg atezolizumab intravenously once every 3 weeks as monotherapy alone or in combination with chemotherapy or anti-angiogenesis, regardless of treatment lines, until the disease progressed. Combination chemotherapy was based on platinum doublet chemotherapy, included etoposide, irinotecan in combination with platinum, paclitaxel / nab-paclitaxel, and gemcitabine. Bevacizumab was used as a combination anti-angiogenic drug. Anlotinib or apatinib were used as targeted drugs.

The first-line therapy is immunotherapy combined with etoposide plus platinum ± anti-angiogenic drug.

The second-line therapy (≥ 2) is immunotherapy ± Chemotherapy with changed protocol ± anti-angiogenic drug.

Exclusion criteria were: (1) patients previously treated with anti-PD-L1/PD-1 drugs; (2) previous or concomitant other malignant tumors; (3) patients used large doses of antibiotics within 1 month.

Detailed patient information, mainly including age at the time of treatment, gender, stage, Eastern Cooperative Oncology Group performance status (ECOG-PS), smoking history, number of distant metastases, treatment regimen, comorbidities, number of treatment lines, whether or not they had received radiotherapy, and the date of death or last follow-up were collected through electronic medical records or telephone follow-up. The comorbidity was diagnosed when any of the following conditions were identified: chronic obstructive lung disease (COPD), bronchiectasis, cardiovascular diseases, hypertension, diabetes, gastritis/gastroesophageal reflux disease and renal insufficiency.

Information on concomitant medications at the start of immunotherapy and during treatment were also collected: PPIs, corticosteroids (dose ≥ 10 mg prednisone equivalent per day, with a minimum 24 h of dosing), angiotensin-converting enzyme inhibitors (ACEI), acetylsalicylic acid (low-dose daily for cardiovascular prevention).

### Assessment

The clinical outcomes of interest were the objective response rate (ORR), progression-free survival (PFS) and overall survival (OS). Specifically, the ORR was defined as the portion of patients experiencing an objective response (complete or partial response) as the best response to immunotherapy according to Response Evaluation Criteria in Solid Tumors (RECIST) v1.1 [29]. Adverse events (AEs) were evaluated according to the Common Terminology Criteria for Adverse Events (version 5.0) [30]. PFS was defined as the time from initiation of immunotherapy to disease progression or death, whichever occurred first. OS was defined as the time from initiation of immunotherapy to death. For PFS and for OS, patients without events were considered censored at the time of the last follow-up. Follow-up was carried out until the end of May 15th, 2022.

### Statistical analysis

Baseline patient characteristics were reported with descriptive statistics. Fisher’s exact test or the χ^2^ test was used to analyze clinical variables and ORR. The PFS and OS curves were estimated using the Kaplan-Meier methodology, and the log rank test was used for their univariate analyses. Cox proportional hazards regression was used for the multivariate analyses of PFS and OS and to compute the hazard ratios (HRs) for disease progression and death with 95% confidence intervals (CIs). The variables with *P* < 0.1 identified in univariable analysis were selected for the multivariable analysis. *P* < 0.05 was considered statistically significant. All tests were bidirectional. SPSS statistical software (version 25.0; IBM Corporation, Armonk, NY) was used for data analysis and graphing.

## Results

### Patient characteristics

A total of 208 patients with advanced SCLC (161 male and 47 female) were treated with durvalumab or atezolizumab. The median age was 61 years (range: 28–81 years), and 119 (57.2%) were smokers. Seventy-seven patients had brain metastases (37.0%) and fifty-three patients had liver metastases (25.5%). Among these patients, 107 were first-line treatment, including 102 received anti-PD-L1 treatment combined with chemotherapy, 2 received anti-PD-L1 treatment combined with anti-angiogenesis treatment after chemotherapy, and 3 cases received immune monotherapy after chemotherapy. Among 101 patients who received ≥ 2 line therapy, 64 received anti-PD-L1 therapy combined with chemotherapy, 15 received anti-PD-L1 therapy combined with anti-angiogenesis therapy, 9 received both, and 13 received immunomonotherapy. During the treatment, the incidence of treatment adverse events caused by level 2 treatment accounted for 26%. Overall, 101 (48.6%) participants used PPIs, 41 (40.6%) pantoprazole, 35 (34.7%) lansoprazole, 15 (14.8%) omeprazole, 9 (8.9%) rabeprazole and 1 (1.0%) esomeprazole. Other patient characteristics are presented in Table 1.

**Table 1:** Baseline characteristics of patients divided into those treated with (*n* = 101) and without (*n* = 107) PPIs

Characteristics		Not used PPIs (*n* = 107)	Used PPIs (*n* = 101)	*P* Value
Age (years)	Median (IQR)	62(56–68)	61(55–67)	0.854
Gender (n, %)	Male	84 (78.5)	77 (76.2)	0.696
	Female	23(21.5)	24 (23.8)	
Smoking (n, %)	No	46 (43.0)	43 (42.6)	0.952
	Yes	61 (57.0)	58 (57.4)	
ECOG PS (n, %)	0–1	45 (42.1)	38 (37.6)	0.514
	≥ 2	62 (57.9)	63 (62.4)	
Radiotherapy (n, %)	No	19 (17.8)	27 (26.7)	0.119
	Yes	88 (82.2)	74 (73.3)	
Lines of therapy (n, %)	1	50 (46.7)	57 (56.4)	0.162
	≥ 2	57 (53.3)	44 (43.6)	
Distant organ metastasis	M1a	31 (29.0)	21 (20.8)	0.173
	M1b/c	76 (71.0)	80 (79.2)	
Brain metastases	No	66 (61.7)	65 (64.4)	0.690
	Yes	41 (38.3)	36 (35.6)	
Liver metastases	No	87 (81.3)	68 (67.3)	0.021
	Yes	20 (18.7)	33 (32.7)	
Combination regimen	No	14 (13.1)	2 (2.0)	< 0.001
	Chemotherapy	76 (71.0)	90 (89.1)	
	Anti-angiogenic therapy	14 (13.1)	3 (3.0)	
	Both	3 (2.8)	6 (5.9)	
AEs (n, %)	No/1	84 (78.5)	70 (69.3)	0.130
	2	23 (21.6)	31 (30.7)	
Comorbidities (n, %)	No	54 (50.5)	40 (39.6)	0.116
	Yes	53 (49.5)	61 (60.4)	
Corticosteroids (n, %)	No	76 (71.0)	39 (38.6)	< 0.001
	Yes	31 (29.0)	62 (61.4)	
Acetylsalicylic acid (n, %)	No	85 (79.4)	78 (77.2)	0.699
	Yes	22 (20.6)	23 (22.8)	
ACEIs (n, %)	No	83 (77.6)	63 (62.4)	0.017
	Yes	24 (22.4)	38 (37.6)	
Best response	CR + PR	59 (55.1)	62 (61.4)	0.361
	SD + PD	48 (44.9)	39 (38.6)	

Proton pump inhibitors (PPIs); Eastern Cooperative Oncology Group performance status (ECOG PS), Adverse events (AEs), Angiotensin-converting enzyme inhibitors (ACEIs)

M1a refers to separate tumor nodule(s) in a contralateral lobe; tumor with pleural or pericardial nodules or malignant pleural or pericardial eusion; M1b/c refers to extrathoracic metastases in a single organ or in multiple organs

### Clinical outcomes of anti-PD-L1 therapy

The median OS time was 21.6 months (95% CI: 18.5–24.7); follow-up ended on May 15th, 2022. The median PFS time of all patients treated with durvalumab or atezolizumab was 8.2 months (95%CI: 7.0-9.4; 167 progression events) respectively (Fig. S1). One hundred and twenty-one (58.2%) cases reached CR or PR, and eighty-seven (41.8%) reached SD or PD. (Table 1)

### Univariate and multivariate analyses

**Fig. 1:**
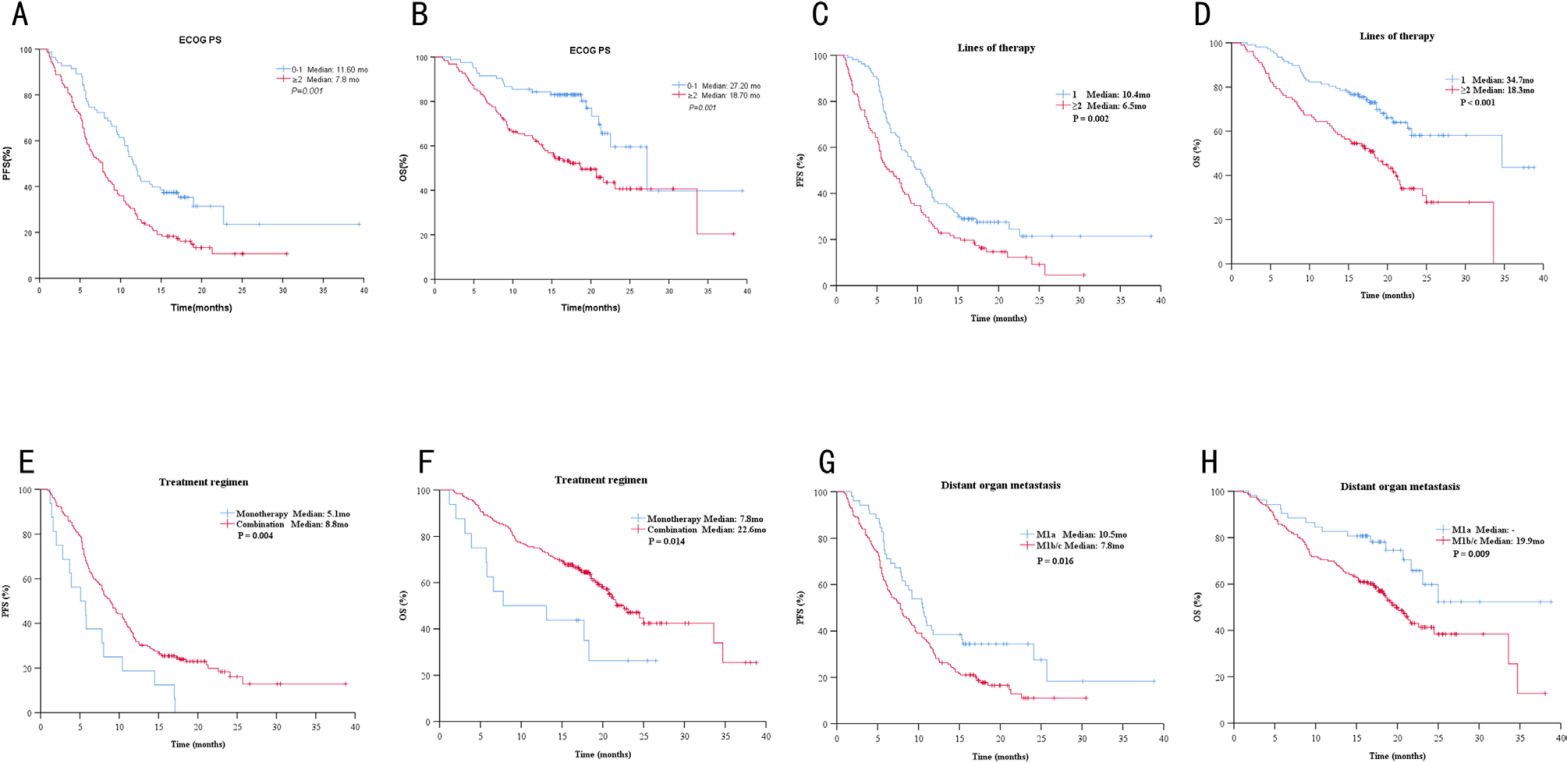
Kaplan–Meier curves for PFS and for OS according to patients’ characteristics at baseline. Kaplan-Meier curves for PSF **(A)** and OS **(B)** grouped by ECOG-PS. Kaplan-Meier survival for PSF **(C)** and OS **(D)** grouped by treatment line. Kaplan-Meier curves for PSF **(E)** and OS **(F)** grouped by treatment regimen. Kaplan-Meier curves for PSF **(G)** and OS **(H)** grouped by distant organ metastasis

**Fig. 2:**
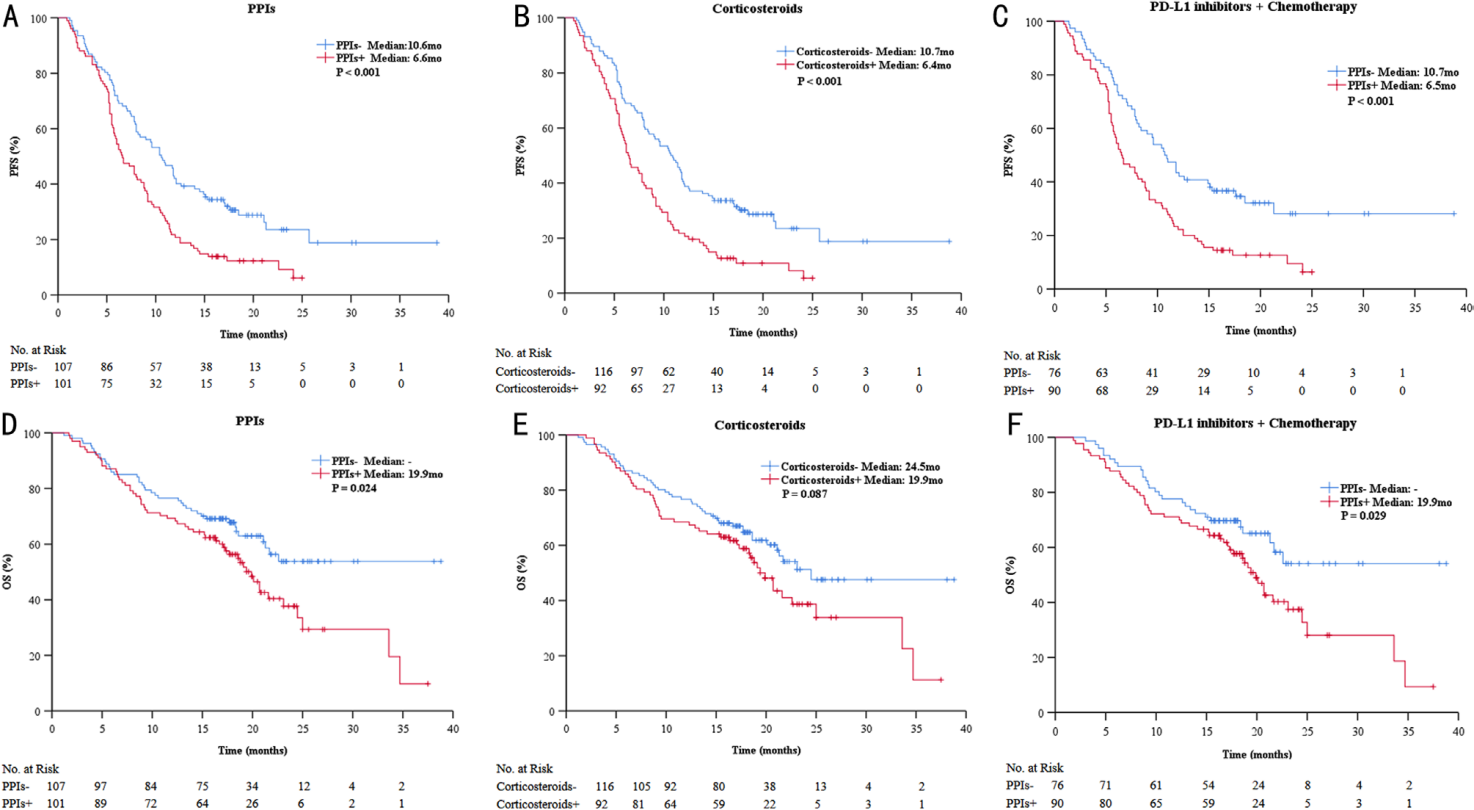
Kaplan–Meier curves for PFS and for OS among patients treated with PD-L1 inhibitors. Kaplane-Meier curves for progression-free survival (PFS) of patients with advanced SCLC treated with PD-L1 inhibitors + proton pump inhibitors (PPIs) **(A)**, PD-L1 inhibitors + corticosteroids **(B)** and PD-L1 inhibitors + chemotherapy + PPIs **(C)**. Kaplane-Meier curves for overall survival (OS) of patients with advanced SCLC treated with PD-L1 inhibitors + PPIs **(D)**, PD-L1 inhibitors + corticosteroids **(E)** and PD-L1 inhibitors + chemotherapy + PPIs **(F)**

Univariate analysis revealed that ECOGPS, lines of therapy, treatment regimen, distant organ metastasis, used of PPIs, and used of corticosteroids were significantly associated with PFS and OS (*P* < 0.10, Tables 2 and 3; Figs. 1 and 2).

**Table 2:** Univariate and Multivariate analysis of Clinical factors on PFS

Factors	Univariate Analysis		Multivariate Analysis	
	HR(95%CI)	*P* value	HR(95%CI)	*P* value
Age (< 60/≥60 years)	0.997(0.733–1.355)	0.985	-	-
Gender (Male/Female)	1.315(0.897–1.929)	0.157	-	-
ECOG PS (0–1/≥2)	0.583(0.423–0.803)	0.001	0.656(0.474–0.909)	0.011
Smoking history (Never/Now or ever)	0.864(0.633–1.178)	0.351	-	-
Lines of therapy (1/≥2)	0.618(0.456–0.837)	0.002	0.608(0.440–0.840)	0.003
Treatment regimen (monotherapy/. combination)	2.078(1.239–3.488)	0.004	2.182(1.264–3.765)	0.005
Comorbidities (No/Yes)	0.943(0.695–1.279)	0.704	-	-
Distant organ metastasis (M1a/M1b/c)	0.637(0.440–0.923)	0.016	0.758(0.521–1.104)	0.149
Liver metastases (No/Yes)	1.009(0.708–1.436)	0.962		
PPIs (No/Yes)	1.749(1.285–2.380)	< 0.001	1.7431(1.231–2.073)	0.002
Corticosteroids (No/Yes)	1.831(1.346–2.489)	< 0.001	1.481(1.058–2.073)	0.022
Acetylsalicylic acid (No/Yes)	0.915(0.627–1.334)	0.643	-	-
ACEIs (No/Yes)	1.141(0.821–1.586)	0.429	-	-

Eastern Cooperative Oncology Group performance status (ECOG PS), Proton pump inhibitors (PPIs); Angiotensin-converting enzyme inhibitors (ACEIs). HR Z hazard ratio; CI Z confidence interval

**Table 3:** Univariate and Multivariate analysis of Clinical factors on OS

Factors	Univariate Analysis		Multivariate Analysis	
	HR(95%CI)	*P* value	HR(95%CI)	*P* value
Age (< 60/≥60 years)	0.787(0.525–1.178)	0.243	-	-
Gender (Male/Female)	1.234(0.754–2.021)	0.402	-	-
ECOG PS (0–1/≥2)	0.364(0.277–0.586)	< 0.001	0.379(0.235–0.613)	< 0.001
Smoking history (Never/Now or ever)	0.850(0.569–1.272)	0.430	-	-
Lines of therapy (1/≥2)	0.435(0.287–0.659)	< 0.001	0.461(0.299–0.711)	< 0.001
Treatment regimen (monotherapy/. combination)	2.155(1.149–4.044)	0.014	2.159(1.095–4.257)	0.026
Comorbidities (No/Yes)	0.962(0.646–1.432)	0.847	-	-
Distant organ metastasis (M1a/M1b/c)	0.495(0.289–0.847)	0.009	0.567(0.329–0.975)	0.040
Liver metastases (No/Yes)	0.824(0.531–1.278)	0.385		
PPIs (No/Yes)	1.583(1.059–2.366)	0.024	1.982(1.260–3.118)	0.003
Corticosteroids (No/Yes)	1.411(0.949–2.097)	0.087	1.020(0.669–1.557)	0.926
ACEIs (No/Yes)	0.818(0.525–1.274)	0.373	-	-
Acetylsalicylic acid (No/Yes)	1.480(0.923–2.374)	0.101	-	-

Eastern Cooperative Oncology Group performance status (ECOG PS), Proton pump inhibitors (PPIs); Angiotensin-converting enzyme inhibitors (ACEIs). HR Z hazard ratio; CI Z confidence interval

Multivariate analysis indicated that ECOGPS, lines of therapy, treatment regimen, used of PPIs, and used of corticosteroids were significantly associated with patient PFS, whereas significant associations were not observed between OS and use of corticosteroids (*P* < 0.05, Tables 2 and 3).

### Clinical outcomes of anti-PD-L1 therapy in the subgroups treated or not treated with PPIs, corticosteroids, ACEI, and acetylsalicylic acid

The median PFS time of patients treated with PPIs was 6.6 months (95% CI, 5.2-8.0) and the median PFS time of patients not treated with PPIs was 10.6 months (95% CI, 8.8–12.4). The median OS of patients treated and those not treated with PPIs was 19.9 months (95% CI, 17.6–22.2) and not reached, respectively (Fig. 2A and D). The differences between the survival curves with regard to PFS and OS were statistically significant (*P* < 0.001 and *P* = 0.024, respectively). Concomitant PPI use was associated with 74.9% increased risk of progression [HR = 1.749, 95%CI (1.285–2.380)] and 58.3% increased the risk of death[HR = 1.583, 95%CI (1.059–2.366)].

Although the median PFS time of patients not received corticosteroids treatment was 10.7 months (95% CI, 8.9–12.5), which was significantly longer than the median PFS time of patients receiving corticosteroids treatment (6.4 months; 95% CI, 5.1–7.7), there was no significant difference in the median OS of patients (Fig. 2B and E). And corticosteroids use was associated with a greater risk (83.1%) of progression [HR = 1.831, 95%CI (1.346–2.489)].

There was no significant difference between the survival curves of PFS and OS treated with ACEI or acetylsalicylic acid (*P* > 0.05).

### Clinical outcomes of anti-PD-L1 therapy combined with chemotherapy in the subgroups treated or not treated with PPIs

In the subset analysis, the median PFS time was 6.5 months (95% CI, 5.0–8.0)for PPIs users compared to 10.7 months (95% CI, 8.7–12.7) for PPIs nonusers (*P* < 0.001; Fig. 1C). Similar, median OS of patients treated and those not treated with PPIs was 19.9 months (95% CI, 18.1–21.7) and not reached, respectively (*P* = 0.029, Fig. 2F).

### Clinical outcomes of different therapy lines on the subgroup treated or not treated with PPIs

**Fig. 3:**
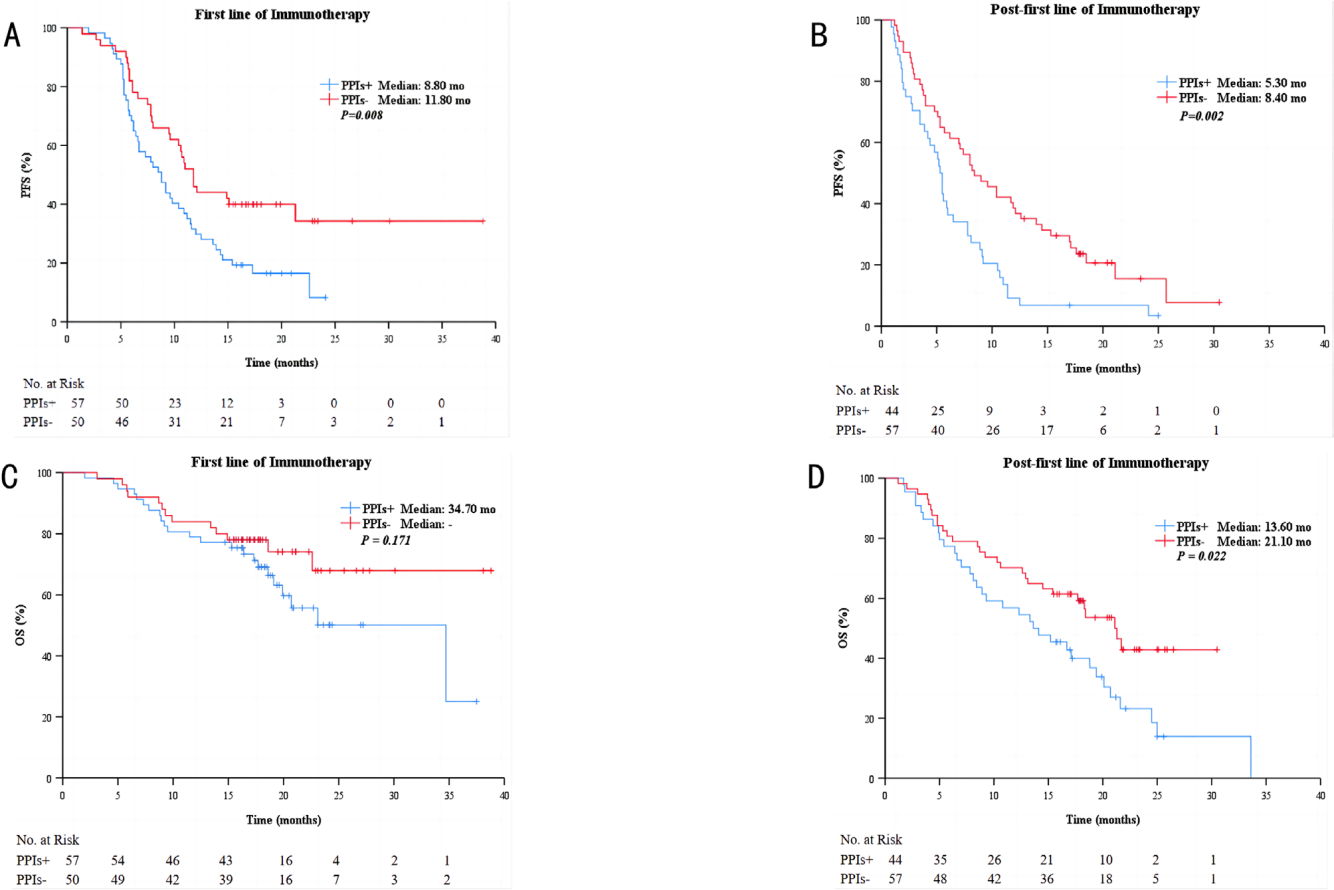
Kaplan–Meier curves for PFS and for OS among patients treated with different lines of therapy. Kaplane-Meier curves for PFS of patients treated with first line of immunotherapy **(A)** and post-first lines of immunotherapy **(B)**. Kaplane-Meier curves for OS of of patients treated with first line of immunotherapy **(C)** and post-first lines of immunotherapy **(D)**

In the first line of immunotherapy subgroup analysis, the median PFS time was 8.8 months (95% CI, 6.8–10.8)for PPIs users compared to 11.8 months (95% CI, 10.2–13.4) for PPIs nonusers (*P =* 0.008; Fig. 3A). The median OS of patients treated and those not treated with PPIs was 34.7months (95% CI, 18.3–51.1) and not reached, respectively (*P* = 0.171, Fig. 3C).

And in the post-first lines of immunotherapy subgroup, the median PFS time was 5.3 months (95% CI, 4.5–6.1)for PPIs users compared to 8.4 months (95% CI, 5.6–11.2) for PPIs nonusers (*P =* 0.002; Fig. 3B). The median OS of patients treated and those not treated with PPIs was 13.6 months (95% CI, 7.4–19.8) and 21.1 months (95% CI, 17.4–24.8), respectively (*P* = 0.022, Fig. 3D).

### Treatment Response of anti-PD-L1 therapy in the subgroups treated or not treated with PPIs, corticosteroids, ACEI, and acetylsalicylic acid

As shown in Table S1 and Fig. S2, there were no correlation between PPIs, corticosteroids, ACEI, acetylsalicylic acid and ORR.

### Progression patterns

**Fig. 4:**
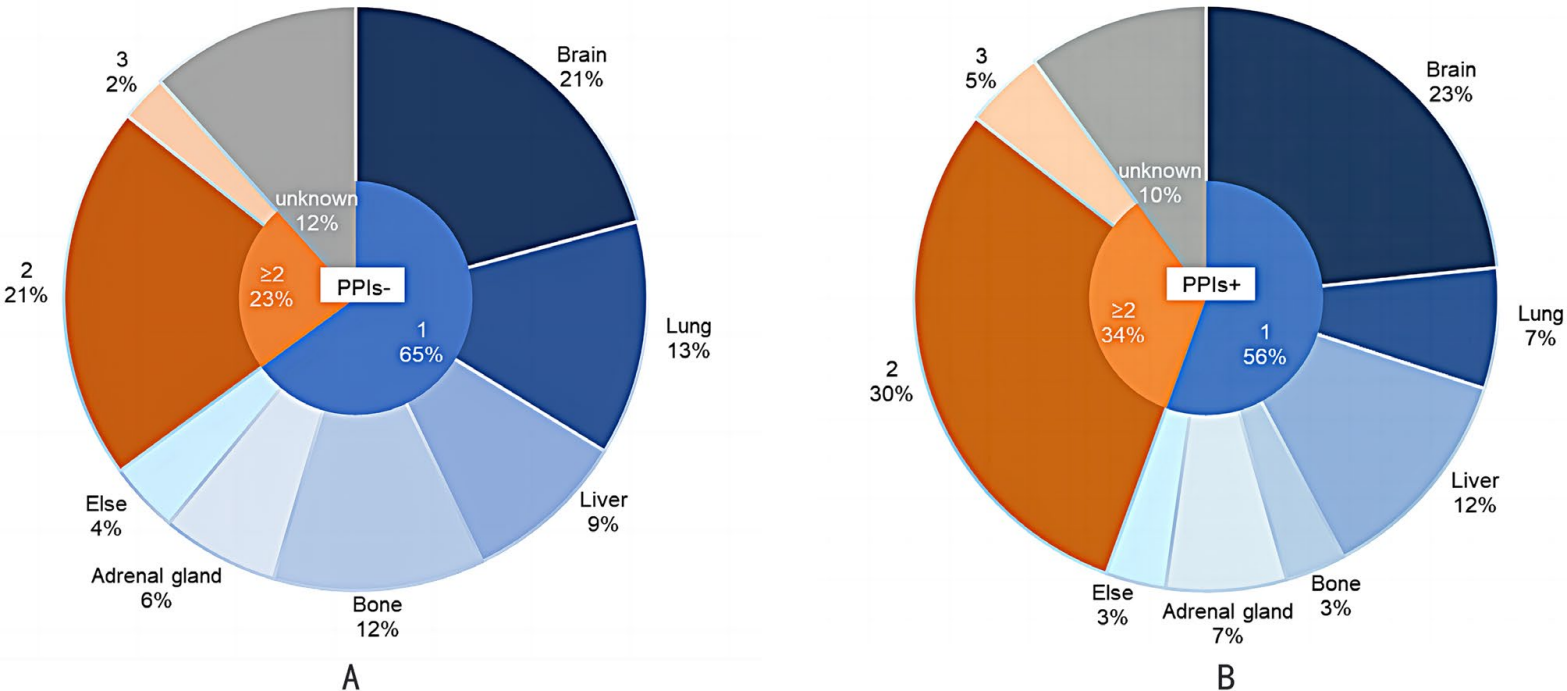
Progression patterns. Pie chart of progression patterns among patients with disease progression in the PPIs- (*n* = 77; **A**) and PPIs+ (*n* = 90; **B**) groups. Blue, one site of disease progression (oligo-progression); orange, more than or equal to two sites of disease progression; grey, site of progression is unknown; PPIs-, treated with PD-L1 inhibitors without proton pump inhibitors; PPIs+, treated with PD-L1 inhibitors plus proton pump inhibitors

In terms of disease progression patterns, patients in the PPIs + group were more likely to possess multiple (≥ 2) progression sites (34% vs. 23%) than patients in the PPIs- group. In the PPIs- group, 50 patients experienced oligo-lesion progression, with the most common progression site being the brain (21%), followed by the lung (13%) and bone (12%) (Fig. 4). Of the 50 patients (56%) in the PPIs + group with definite progression sites, the most common progression sites were the brain (21 patients, 23%) and liver (11 patients, 12%), with 6 cases each experiencing pulmonary (7%) or adrenal gland (7%) progression.

### Clinical outcomes of patients with or without liver metastasis

Univariate analysis showed that liver metastasis was not significantly associated with PFS and OS in patients receiving immunotherapy (*P* > 0.10, Tables 2 and 3 and Fig. S3). However, in patients without liver metastasis, PFS treated with PPIs in immunotherapy were significantly worse than PFS treated without PPIs, at 6.4 months (95%CI, 4.8-8.0) and 11.0 months (95%CI, 9.0–13.0), respectively (*P* = 0.002), while PFS was not significantly different in patients with liver metastasis (*P* = 0.096, Fig. S3).

## Discussion

Chemotherapy combined with PD-L1 immunotherapy has become the new standard first-line treatment regimen for extensive stage SCLC by the Food and Drug Administration, due to its substantial survival gains [5–10, 31]. According to the KEYNOTE-028 and KEYNOTE-158 trials, SCLC patients with two or more lines of previous therapy failure have shown clinical benefit with pembrolizumab [32–34]. In addition, nivolumab monotherapy is an effective third- or later-line treatment for recurrent SCLC [35]. This present study performed a retrospective analysis to examine the influence of PPIs on the clinical outcomes of advanced SCLC patients treated with durvalumab or atezolizumab. Nearly 80% of patients received chemotherapy combined with PD-L1 immunotherapy. The median PFS time for all patients receiving PD-L1 treatment was 8.2 months (95%CI:7.0-9.4; 167 progression events). It was also worth noting that patients with lower ECOG PS scores and first-line ICIs treatment can also achieve better clinical outcomes and prognosis. The ORR was up to 72.0% after the first-line PD-L1 treatment, which was increased by 28.4% compared with the second-line treatment of ORR. The MOUSEION-01 study emphasized that there is a certain association between sex and outcome in cancer patients receiving a combination of immunotherapy and immuno-oncology therapy. Although there was no gender difference in our study, high quality trials of potential confounders are needed [36].

Currently, PPIs were often used as long-term medications in patients with cancer to treat and prevent gastrointestinal discomfort symptoms and gastric mucosal damage [37]. Nearly half (48.6%) of the patients used PPIs when treated with PD-L1 inhibitors in our study. We showed that the use of PPIs with PD-L1 inhibitors was associated with a greater risk of progression or death. Also in the subgroup of immunocombination chemotherapy, using PPIs had adverse effects on the efficacy of ICIs, with patients having a shorter PFS as well as OS. Similarly, the Baek et al. [38] study on 1646 advanced NSCLC patients found a 28% increased risk of mortality when using PPIs compared with no PPIs [HR = 1.28, 95%CI (1.13–1.46)]. However, it is noteworthy that the meta-analysis of Liu et al. [39] showed that PPIs use was associated with reduced PFS in NSCLC patients, but not in melanoma, suggesting that the effect of PPIs on ICIs efficacy may be related with the type of cancer. Much recent research has revealed that PPIs use may be associated with poor prognosis in lung cancer patients treated with ICIs therapies, so the PPIs should be carefully used either before or during ICIs treatment.

Unlike the mechanism that antibiotics directly affect intestinal flora [40–43], PPIs may indirectly affect the number and type of intestinal flora through the following reasons: (1) inhibit gastric acid secretion to change the pH level of the intestine [44, 45]. (2) It interferes with nutrient absorption by inducing hormone changes and changing the bacterial decomposition mode of substrate through a pH independent mechanism [46]. (3) Alternatively, PPIs may directly suppress the immune system through the exertion of anti-inflammatory effects by reducing the secretion of pro-inflammatory cytokines and adhesion molecules expressed by inflammatory cells [47, 48]. Although the above mechanism is a potential explanation for this association, the underlying mechanisms that govern the existence of this phenotype remain unclear. It is urgent that future research should focus on elucidating the possible mechanism for interactions of ICIs with co-medications, and the role of the microbiome.

Corticosteroids are essential in cancer care [49], and are often used in the treatment of AEs to relieve patients’ symptoms. In this study, there was no ≥ 3 grade AEs, which can be considered that immunotherapy is feasible, safe and well tolerated in SCLC patients. However, long-term corticosteroids during PD-L1 immunotherapy suggest shorter PFS, and have no significant effect on OS. Thompson et al. [50] found that the use of high-dose corticosteroids in the treatment of gastroenterocolitis caused by ICIs suggests shorter PFS. It is likely that corticosteroids lead to T-cell down-regulation and apoptosis, changing the peripheral blood CD8 / Treg cell ratio to attenuate the antitumor effect of ICIs [51, 52]. To date, there were few SCLC information about the effect of corticosteroids on ICIs efficiency. Hence, further large-scale prospective cohort studies are warranted.

Interestingly, the progression pattern analysis conducted in this study revealed that most patients with SCLC had single organ progression (59.9%) and that the untreated of PPIs to cotherapy reduced the rate of multisite (≥ 2 sites) progression. Notably, most patients had brain progression, with similar patterns of progression observed among the two groups. The failure of the original pulmonary disease treatment likely occurred in distant metastasis of SCLC. Thoracic radiotherapy, whole-brain radiation therapy and prophylactic cranial irradiation, are considered optional in the most recent current guidelines for the treatment of extensive stage SCLC [53, 54]. Radiotherapy, as an ideal partner for ICIs, appears to enhance the anti-tumor response by altering the tumor microenvironment [55]. In addition, liver metastasis should be valued in the used of PPIs to cotherapy. The results of the IMPower133 study showed that extensive stage SCLC patients without liver metastases benefited more from immunotherapy [56]. The progression of liver metastasis may have influenced ICIs resistance, contributing to poor survival [57]. These factors are recognized as being associated with poor PFS, as well as in other, smaller, real-world evidence studies [58, 59]. However, in our study, PFS was not significantly associated with the presence or absence of liver metastasis in patients receiving immunotherapy. Interestingly, the used of PPIs during immunotherapy produced poorer PFS in patients without liver metastasis, while PFS in patients with liver metastasis had no effect. At the same time, liver metastases often occur during disease progression, especially in patients treated with PPIs.

This study has the following limitations. Firstly, this is a retrospective study conducted in a single institution with a relatively small sample size. There are relatively a few patients who use antibiotics under various circumstances during continuous immunotherapy, and the impact of this on the changes of intestinal microbiota needs research in the future. Secondly, further evaluation of the dosage of PPIs and corticosteroids is needed to confirm the effect of a series of changes on. Thirdly, there is no separate distinction between durvalumab or atezolizumab and the impact of using PPIs. Finally, this study only analyzed the relationship between the baseline organ metastasis and the patient’s OS, and the recurrence and metastasis in the treatment of SCLC patients may also affect the survival of patients. However, most current studies are small and underpowered; larger, prospective clinical trials are highly needed to address this unmet need [21]. As the number of patients receiving ICIs and SCLC is further increasing, determining the impact of these agents on SCLC immunotherapy is an urgent need for this aggressive malignancy.

## Conclusion

In conclusion, this study demonstrated that PPIs use during PD-L1 inhibitors treatment initiation was correlated with decreased PFS and OS, which have a negative impact on the clinical outcomes of patients with advanced SCLC. The findings also need larger prospective studies while adjusting for other confounding factors and evaluating patient survival and changes in intestinal microbiota affected by PPIs use.

### Electronic supplementary material

Below is the link to the electronic supplementary material.

Supplementary Material 1

Supplementary Material 2

## Data Availability

The raw data supporting the conclusions of this article will be made available by the corresponding authors.

## List of abbreviations

PD: L1 Programmed death-ligand 1
SCLC: Small cell lung cancer
PPIs: Proton pump inhibitors
PFS: Progression-free survival
OS: Overall survival
ECOG-PS: Eastern Cooperative Oncology Group performance status
COPD: Chronic obstructive lung disease
ACEI: Angiotensin-converting enzyme inhibitors
ORR: Objective response rate
AEs: Adverse events
