# Supplementary material for: PPIs therapy has a negative impact on the clinical outcomes of advanced SCLC patients treated with PD-L1 inhibitors
Source: BMC Pulm Med. 2023 Nov 11;23:438. doi: 10.1186/s12890-023-02754-4 (PMC10638834; doi:10.1186/s12890-023-02754-4)
Supplement: Supplementary file 1 — Supplementary Material 1 [file 12890_2023_2754_MOESM1_ESM.docx]

**Table S1**  Response Rate in the subgroups

| Subgroup  (n, %) | | CR+PR  (n=121) | SD+PD  (n=87) | P Value |
| --- | --- | --- | --- | --- |
| ECOG PS | 0-1 | 53 (63.9) | 30 (36.1) | 0.176 |
|  | ≥2 | 68 (54.4) | 57 (45.6) |  |
| Lines of therapy | 1 | 79 (73.8) | 28 (26.2) | <0.001 |
|  | ≥ 2 | 42 (41.6) | 59 (58.4) |  |
| Treatment regimen | Monotherapy | 0 (0) | 16 (100) | <0.001 |
|  | Combination | 121 (63.0) | 71 (37.0) |  |
| Distant organ metastasis | M1a | 32 (61.5) | 20 (38.5) | 0.570 |
|  | M1b/c | 89 (57.1) | 67 (42.9) |  |
| PPIs | No | 59 (55.1) | 48 (44.9) | 0.361 |
|  | Yes | 62 (61.4) | 39 (38.6) |  |
| Corticosteroids | No | 66 (56.9) | 50 (43.1) | 0.675 |
|  | Yes | 55 (59.8) | 37 (40.2) |  |
| Acetylsalicylic acid | No | 96 (58.9) | 67 (41.1) | 0.688 |
|  | Yes | 25 (55.6) | 20(44.4) |  |
| ACEIs | No | 80 (54.8) | 66 (45.2) | 0.130 |
|  | Yes | 41 (66.1) | 21 (33.9) |  |

Objective response rate (ORR) was defined as the portion of patients experiencing an objective response (complete or partial response) as the best response to immunotherapy according to Response Evaluation Criteria in Solid Tumors (RECIST) v1.1.

Complete response (CR); Partial response (PR); Stable disease (SD); Progressive disease (PD).
