# Supplementary material for: PPIs therapy has a negative impact on the clinical outcomes of advanced SCLC patients treated with PD-L1 inhibitors
Source: BMC Pulm Med. 2023 Nov 11;23:438. doi: 10.1186/s12890-023-02754-4 (PMC10638834; doi:10.1186/s12890-023-02754-4)
Supplement: Supplementary file 2 — Supplementary Material 2 [file 12890_2023_2754_MOESM2_ESM.docx]

**Figure S1** Kaplan–Meier curves for PFS and for OS.

**Figure S2** Treatment Response of anti-PD-L1 therapy in the subgroups

**Figure S3** Kaplan–Meier curves for PFS and for OS among patients with or without liver metastasis.
